# Different Chinese herbal medicine therapy for idiopathic thrombocytopenic purpura: A protocol for systematic review and Bayesian network meta-analysis

**DOI:** 10.1097/MD.0000000000025341

**Published:** 2021-04-02

**Authors:** Wen-Ting Chen, Rui-Mei Tang, Ying Huang, Yan-Ping Pan, Shu-Wen Wang, Gu-Yun Wang

**Affiliations:** Department of Hematology, Hainan General Hospital (Hainan Affiliated Hospital of Hainan Medical University), Xiuying District, Haikou City, Hainan Province, China.

**Keywords:** Bayesian network meta-analysis, Chinese herbal medicine, clinical effectiveness, idiopathic thrombocytopenic purpura, safety

## Abstract

**Background:**

Idiopathic thrombocytopenic purpura (ITP) is a common immune system and blood system disease in clinical practice, and it is a hemorrhagic disease caused by immune factors causing platelet destruction and decreasing number of platelets. There is currently no effective treatment plan for ITP. At this stage, glucocorticoid and gamma globulin are mostly used in the treatment of ITP, and some patients use splenectomy to achieve therapeutic purposes, but the various treatment methods are inadequate. At this stage, a large number of randomized controlled studies have reported that Chinese herbal medicine has achieved good curative effect in the treatment of ITP. However, due to the variety of Chinese herbal medicine, there has been no evidence of the effectiveness and safety of Chinese herbal medicine in the treatment of ITP. Because of the above reasons, this study uses the network meta-analysis method based on Bayesian method to compare the clinical efficacy and safety of different kinds of Chinese herbal medicine in the treatment of ITP through direct and indirect comparison, in order to provide evidence-based medical support for the treatment of ITP with Chinese herbal medicine.

**Methods:**

This study uses the method of combining free words with theme words, and using computer to retrieve PubMed, EMbase, The Cochrane Library, WANFANG Database, CNKI, and VIP Database, etc, to collect the randomized control studies on Chinese herbal medicine in the treatment of ITP. The retrieval time is from the establishment of each database to January 2021, and the retrieval languages are Chinese and English. Two researchers independently read the title, abstract and full text of the article to determine whether it is included in the literature; In the event of a disagreement, a third researcher will join the discussion to resolve the disagreement; For the literature that lacks information, trying to contact the original author of the document to supplement it. The literature quality evaluation carried out by using the Stata 14.0 software to draw network and funnel plots, in accordance with the quality evaluation criteria of version 5.1.0 of the Cochrane System Evaluation Manual. Statistical analysis is performed by using ADDIS 1.16.8 software based on the Bayesian model.

**Results:**

This study will compare the clinical efficacy and safety of different types of Chinese herbal medicine in the treatment of idiopathic thrombocytopenic purpura through the method of network meta-analysis, and rank the different types of Chinese herbal medicine according to their effectiveness, and the results will be published in a peer-reviewed, high-quality academic journal.

**Conclusion:**

This study will find effective and safe Chinese herbal medicine for clinical treatment of ITP from the perspective of evidence-based medicine, and benefit more ITP patients.

## 1 Introduction

Idiopathic thrombocytopenic purpura also known as immune thrombocytopenic, and it is a hemorrhagic disease caused by platelet excessive destruction by a group of immune-mediated.^[1]^ It is characterized by extensive skin mucosa and visceral bleeding, peripheral thrombocytopenia, shortened platelet lifespan, bone marrow megakaryocyte development and maturation disorder, and the appearance of platelet glycoprotein specific autoantibodies.^[2]^ ITP is the most common thrombocytopenic purpura, the current incidence rate is increasing year by year. The onset of adult is more hidden and chronic, with the majority of women in childbearing age, and the incidence rate of elderly people is on the rise. Although the disease is not malignant, it is prone to recurrence. The probability of fatal bleeding and the 2 years mortality rate in patients with a platelet count continuously below 30 × 10^9^/L is 4 times that of the general population.^[3]^ The fear of severe bleeding, adverse drug reactions and expensive medical expenses can easily cause multiple psychological, physical, and economic stress on patients, seriously affecting their quality of life. Therefore, it is of great significance to evaluate the safe and effective treatment and effectiveness of the disease. Glucocorticoid is currently the drug of choice for ITP treatment. However, the long-term use can cause glucocorticoid tolerance or dependence in some patients, as well as side effects such as blood pressure, elevated blood sugar, immune disorders, femoral head necrosis, etc.^[4]^

There is no ITP disease name in ancient Chinese medical books. Its clinical symptoms are mainly bleeding, so it belongs to the category of “blood syndrome.”^[5]^ Chinese herbal medicine has a long history in the treatment of ITP. At present, the treatment of ITP by Chinese herbal medicine has been widely used in clinical practice. The thoughts of therapy with syndrome differentiation of Chinese herbal medicine and the characteristics of low side effects of Chinese herbal medicine have shown very good curative effects in the treatment of this disease. At present, the consensus has been reached that the patients are divided into acute attack stage, protracted stage, and recovery stage. The syndromes are classified into “Yin deficiency inner heat syndrome,” “deficiency of vital energy” and “splenic failure to control blood,” et al. Therefore, different Chinese herbal medicine should be used for treatment at different stages.^[6,7]^ Although a large number of randomized controlled studies have reported that Chinese herbal medicine has achieved good efficacy in the treatment of ITP. However, most of them are direct comparisons between Chinese herbal medicine and Western medicines. However, due to the different types of Chinese herbal medicine, there is no evidence-based medicine for direct and indirect comparisons between different types of Chinese herbal medicine.^[8,9]^ This study uses a network meta-analysis based on the Bayesian method. Through direct and indirect comparisons, the clinical efficacy, and safety of different types of Chinese herbal medicine for the treatment of ITP are compared, and the different Chinese herbal medicine are ranked according to the clinical efficacy. The purpose of this study is to provide evidence-based medical evidence support for the treatment of ITP with Chinese herbal medicine. It also provides help for clinicians to choose suitable treatment drugs.

## 2 Methods

### 2.1 Protocol registration

OSF Registration number: DOI 10.17605/OSF.IO/UDKN6 (available from: https://osf.io/udkn6).

### 2.2 Inclusion and exclusion criteria

#### 2.2.1 Types of studies

The randomized control studies on Chinese herbal medicine in the treatment of ITP. The language range is limited to Chinese and English.

#### 2.2.2 Types of participant

All patients met the ITP diagnostic criteria. The patient's age, gender, race, region, and duration of illness are not limited.

#### 2.2.3 Inclusion criteria

1. ITP diagnosis meets the ITP diagnosis criteria established by the *Hemostasis and Thrombosis Group of the Hematology Branch of the Chinese Medical Association in 2009* and excludes secondary thrombocytopenic purpura.^[10]^
2. It conforms to the diagnostic criteria of ITP in the Guiding Principles for Clinical Research of New Chinese herbal medicine.^[11]^
3. Exclude other secondary thrombocytopenia

#### 2.2.4 Exclusion criteria

1. Previous splenectomy treatment.
2. Other diseases that can cause thrombocytopenia.
3. Secondary thrombocytopenia was excluded. Such as aplastic anemia (AA), myelodysplastic syndrome (MDS), HIV infection, drug-induced thrombocytopenia, and other secondary immune thrombocytopenia.
4. Control measures are blank control, placebo control, or other positive controls.
5. Non-randomized controlled studies such as animal experiments, case reports, and reviews.

#### 2.2.5 Experimental interventions and control interventions

*Control group:* ITP was treated with Western medicine alone (the drugs are taken orally), blank control group, placebo group.

*Experimental group:* ITP was treated with Chinese herbal medicine.

Chinese herbal medicine includes traditional formulations, Chinese patent medicines, powder, pills, decoction, etc, but it does not include Chinese herbal medicine injections. Both the experimental group and the control group can be combined with conventional basic treatment, and the dose and course of treatment are not limited. In order to reduce the impact on the target drug, all combination drugs are excluded.

### 2.3 Types of outcome measures

#### 2.3.1 Primary outcomes

1. Total effective rate, total effective rate = (number of markedly effective cases + number of effective cases)/total number of cases in this group × 100%, markedly effective: platelet count ≥100 × 10^9^/L and no bleeding after treatment. Effective: after treatment, the platelet count is ≥30 × 10^9^/L and is at least 2 times higher than the basal platelet count and no bleeding. Ineffective: after treatment, the platelet count is less than 30 × 10^9^/L, and the bleeding symptoms are not improved or worsened.
2. Recurrence rate. After stopping the drug, the proportion of patients who had a recurrence of the disease among the patients whose previous treatment was effective.

#### 2.3.2 Secondary outcomes

1. Platelet function indexes, including platelet count, platelet haematocrit, and mean platelet volume.
2. Bleeding control time.
3. The time for platelet count to return to normal.
4. Blood cell count, including white blood cells, red blood cells, platelets, hemoglobin, etc.
5. Adverse reactions, including cardiovascular reactions, hypocalcemia, citrate poisoning, allergic reactions, dizziness and nausea, gastrointestinal reactions, etc.
6. The average hospitalization time.

### 2.4 Search strategy

This study uses computer to retrieve PubMed, EMbase, The Cochrane Library, WANFANG Database, CNKI, and VIP Database, etc, to collect the randomized control studies on traditional Chinese herbal medicine in the treatment of ITP. The retrieval time is from the establishment of each database to January 2021. In addition, references are retroactively incorporated into the literature to supplement access to the relevant literature. This study uses the method of combining free words with theme words. Taking the Cochrane Library as an example to show the complete literature search process. The specific retrieval strategy of the literature is shown in Table 1.

**Table 1:** Search strategy using the Cochrane Library.

Number	Search strategy
#1	[(Drugs, Chinese herbal) OR (Chinese drugs, plant) OR (Chinese herbal drugs) OR (Herbal drugs, Chinese) OR (Plant extracts, Chinese) OR (Chinese plant extracts) OR (Extracts, Chinese plant)]:Title/Abstract/Keyword
#2	[(Glucocorticoids) OR (Glucocorticoid) OR (Glucocorticoid effect) OR (Effect, glucocorticoid) OR (Glucorticoid effects) OR (Effects, glucorticoid) OR (Aluminum hydroxide) OR (Sucralfate) OR (Methylprednisolone) OR (Prednisone) OR (Dexamethasone)]:Title/Abstract/Keyword
#3	[(Purpuras, thrombocytopenic) OR (Thrombocytopenic purpura) OR (Thrombocytopenic purpuras) OR (Purpura, thrombopenic) OR (Purpuras, thrombopenic) OR (Thrombopenic purpura) OR (Thrombopenic purpuras)]:Title/Abstract/Keyword
#4	[(Purpura, thrombocytopenic, idiopathic) OR (Idiopathic thrombocytopenic purpura) OR (Idiopathic thrombocytopenic purpuras) OR (Purpura, idiopathic thrombocytopenic) OR (Purpuras, idiopathic thrombocytopenic) OR (Thrombocytopenic purpura, idiopathic) OR (Thrombocytopenic purpuras, idiopathic) OR (Immune thrombocytopenic purpura) OR (Immune thrombocytopenic purpuras) OR (Purpura, immune thrombocytopenic) OR (Purpuras, immune thrombocytopenic) OR (Thrombocytopenic purpura, immune) OR (Thrombocytopenic purpuras, immune) OR (Immune thrombocytopenia) OR (Immune thrombocytopenias) OR (Thrombocytopenia, immune) OR (Thrombocytopenias, immune) OR (Thrombocytopenic purpura, autoimmune) OR (Werlhof disease) OR (Disease, werlhof) OR (Werlhof's disease) OR (Disease, werlhof's) OR (Werlhofs disease) OR (Autoimmune thrombocytopenia) OR (Autoimmune thrombocytopenias) OR (Thrombocytopenia, autoimmune) OR (Thrombocytopenias, autoimmune) OR (Autoimmune thrombocytopenic purpura) OR (Autoimmune thrombocytopenic purpuras) OR (Purpura, autoimmune thrombocytopenic) OR (Purpuras, autoimmune thrombocytopenic) OR (Purpura, thrombocytopenic, autoimmune)]:Title/Abstract/Keyword
#5	#1 OR #2
#6	#3 OR #4
#7	#5 AND #6

### 2.5 Data collection and analysis

#### 2.5.1 Selection of trials

**Figure 1:**
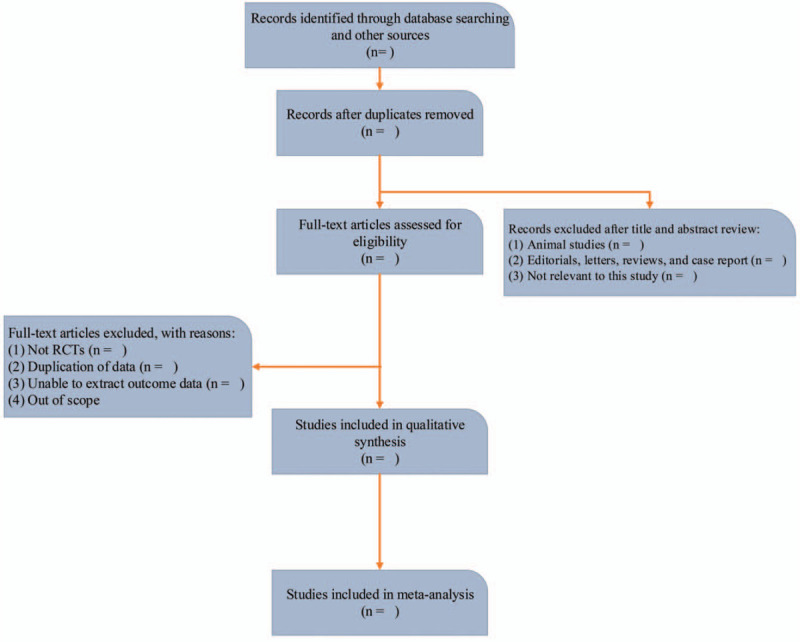
The flow chart of document collection and sorting.

The 2 researchers screen the selected literature separately according to the above-mentioned inclusion and exclusion criteria (Fig. 1). If the Chinese literature does not meet the inclusion criteria, it is directly excluded. For literature that meets the inclusion criteria, on the basis of reading the summary and the full text, it is decided whether to include it in this study. If necessary, contacting the original study author by e-mail or telephone for information that is not certain but is important for this study. If the 2 researchers disagree on whether the selected literature should be included, they may discuss it together. If the differences cannot be resolved after the discussion, a third researcher may be invited to join and an agreement can be reached after the discussion.

#### 2.5.2 Data extraction

According to the purpose and outcome indicators of the study to determine the types and projects included in the study data extraction, developing the relevant Excel table, extracting the main content of the data:

1. the basic characteristics of the included research: the title of the paper, the author of the study, the publication year of the paper and the study area, etc;
2. the type of study included in the study;
3. the basic information on the cases included in the study: the number of patients, the general situation, the intervention mode included in the study groups and control groups, etc;
4. the key factors of evaluation bias;
5. accurate extraction of data from the necessary outcome indicators of this study.

Paying attention in the extraction data: First, the excel data extraction table should be checked, if there are problems found, timely checking the relevant information and data and real-time changes and optimization of the table data. Second, in order to prevent data extraction errors, 2 researchers should independently complete the data extraction work, after the data extraction, 2 researchers together to check the data. If the data is checked for the same and there is no objection, further data analysis will be made. If the data are found to be inconsistent in the process of data extraction and checking, the third researcher should be consulted and discussed to resolve the issue.

#### 2.5.3 Dealing with missing data

In case of missing data or incomplete data reports, we will try to contact the corresponding authors to obtain the original data. If the author cannot be contacted, we will analyze it based on the available data. If the key data in the selected literature is missing, a researcher will contact the original author of the paper by email to obtain relevant data. If the original author of the paper cannot be contacted, the paper will be excluded after group discussion.^[12,13]^

### 2.6 Risk of bias assessment

The risk assessment tool of the Cochrane Collaboration Network divides the risk assessment of bias into 7 aspects according to different sources such as selection, implementation, measurement, follow-up, reporting, etc, namely, random sequence generation, allocation hiding, blinding of researchers and subjects, and blind evaluation of research outcome, completeness of result data, selective reporting of research results, and other bias sources.^[14]^ Performing bias risk assessment on the included literature. According to the quality of the included literature, each evaluation indicator has 3 qualities: low risk, high risk, and unclear risk. Summarizing the results obtained, and producing the corresponding bias risk summary chart and percentage chart.^[15]^

### 2.7 Statistical analysis

#### 2.7.1 Network meta-analyses

Using the Stata 14.0 software to draw network diagrams and funnel plots. Statistical analysis is performed using ADDIS 1.16.8 software based on the Bayesian model. The software uses the Markov Chain–Monte Carlo (MCMC) method to a priori and evaluate the data based on the Bayesian framework for Network meta-analysis. The initial iteration is set to 50,000 times. Comparing the deviance information criterion (DIC) between the random effect model and the fixed effect model to judge the fit of the model. The odds ratio of the second classification effect is used, and the mean difference of the continuity effect is a statistic, all using a 95% confidence interval. The level of meta-analysis is set to α = 0.05. The obvious clinical heterogeneity is treated by methods such as subgroup analysis or sensitivity analysis, or only descriptive analysis. Network meta-analysis uses a consistency model, and *P* < .05 is statistically significant. The insistency test uses a node analysis model, and if *P* > .05, it means that there is no evidence to prove the inconsistency between direct comparison and indirect comparison. The convergence of network meta is tested using potentialscale reduced factor. When the PSRF value is closer to 1, the better the convergence result, the better the consistency model, the more credible the conclusion, and vice versa, the lower the credibility. The results are sorted according to Bayesian statistical methods, and the probability of interventions becoming the best treatment is determined based on the surface under the cumulative ranking.^[16]^

#### 2.7.2 Sensitivity analysis

Each time a study is deleted, and then merged separately, while observing the change in the effect amount. If there is a difference between the new merged result and the original statistical result after deleting a study, it is considered that this study has a great impact on the overall effect quantity. Whether it is deleted or not, it needs to be evaluated and discussed.

### 2.8 Publication bias

A comparison-correction funnel plot is drawn with the most recent effectiveness of the largest number of included studies as the outcome index. The dots with different colors in the plot indicated the direct comparison between the 2 interventions. The distribution of funnel plot is basically symmetrical, indicating that the possibility of publication bias or small sample effect is small.

### 2.9 Ethics and dissemination

The literature collected by the Institute is derived from published academic literature in a professional network database, and the data used in statistical analysis can be obtained from these publicly published papers, so the study does not require ethical approval.

### 2.10 Confidence in cumulative evidence

Using the GRADE evaluation system to evaluate the evidence quality of outcome indicators, the quality of the randomized controlled studies included is evaluated from 5 aspects (bias risk, consistency, results, indirectness, accuracy of research, and publication bias), and the evidence quality is divided into “high, medium, low and very low,” and the recommended strength is divided into “strong recommendation and weak recommendation.”^[17,18]^

## 3 Discussion

ITP is an acquired autoimmune hemorrhagic disease. According to incomplete statistics, it accounts for about one third of the total number of hemorrhagic diseases. It is a common hemorrhagic disease characterized by peripheral thrombocytopenia, normal or increased number of megakaryocytes in bone marrow, or accompanied by dysmaturation. Clinically, patients usually have ecchymosis on the skin or mucous membrane. More serious patients may have visceral bleeding or even intracranial hemorrhage, and the risk of bleeding increases with the age of patients. It is of great significance to actively explore the treatment of ITP.

At present, the exact cause of ITP has not been fully elucidated in modern medicine. According to the incidence of ITP, researchers can divide it into acute phase and remission phase. Most scholars believe that there is a correlation between viral infection and acute ITP, but its specific pathogenesis is not clear. In addition, studies have shown that viral antigens can combine with corresponding antibodies to form immune complexes, which can be deposited on platelets and megakaryocytes, so that platelets and megakaryocytes can be eliminated as target tissues.^[19]^ In recent years, studies have found that viruses can still change the structure of platelet membrane glycoproteins, leading to the formation of anti-platelet antibodies, which can mediate complement and monocyte-macrophage destruction of platelets. Therefore, preventing the excessive destruction of platelets and promoting platelet production have become the key research direction of modern treatment of ITP.^[20]^

At present, for the treatment of ITP, Western medicine mainly has the following treatment measures, such as the application of glucocorticoid, platelet transfusion, gamma globulin, the use of immunosuppressants, splenectomy, etc, of which about 30% of the patients have no obvious treatment effect.^[21]^ Some patients with ITP who have been relieved by drug treatment still have problems such as large side effects, high price, and easy recurrence of the treatment drugs, and some patients are difficult to adhere to for a long time. Long term clinical practice has proved that, Chinese herbal medicine has certain advantages in the treatment of modern refractory diseases. Traditional Chinese herbal medicine in the treatment of ITP can effectively alleviate the clinical symptoms of patients, fundamentally improve the immune imbalance of patients, and has less adverse reactions. However, the current shortcoming is the lack of evidence for comparison between different types of Chinese herbal medicine, while traditional meta-analysis can only be used to directly compare 2 interventions. When there is a lack of evidence for direct comparison of 2 interventions, the traditional meta-analysis method. They cannot be compared and evaluated, and traditional meta-analysis cannot compare multiple interventions at the same time. The development of network Meta provides the possibility to solve the above situation. It can combine the direct comparison evidence and indirect comparison evidence between different interventions in clinical trials for meta-analysis, so as to compare the advantages and disadvantages of multiple clinical interventions. And ranking, to provide guidance for clinical selection of appropriate treatment methods. Therefore, this study uses the network meta-analysis method based on Bayesian method to compare the clinical efficacy and safety of different kinds of Chinese herbal medicine in the treatment of ITP through direct and indirect comparison, in order to provide evidence-based medical support for the treatment of ITP with Chinese herbal medicine.

## Author contributions

**Conceptualization:** Yan-Ping Pan, Gu-Yun Wang.

**Data curation:** Wen-Ting Chen, Rui-Mei Tang.

**Formal analysis:** Wen-Ting Chen, Rui-Mei Tang, Ying Huang, Yan-Ping Pan.

**Funding acquisition:** Gu-Yun Wang.

**Resources:** Wen-Ting Chen, Rui-Mei Tang, Ying Huang, Shu-Wen Wang.

**Software:** Wen-Ting Chen, Rui-Mei Tang, Yan-Ping Pan, Shu-Wen Wang.

**Supervision:** Wen-Ting Chen.

**Writing – original draft:** Wen-Ting Chen, Rui-Mei Tang, Ying Huang, Yan-Ping Pan, Shu-Wen Wang.

**Writing – review & editing:** Gu-Yun Wang.

## References

[1] Li SS Jiang H. Advances in pathogenesis of chronic idiopathic thrombocytopenic purpura. Int J Pediatr 2014;41:534–7.

[2] Semple JW Rebetz J Maouia A. An update on the pathophysiology of immune thrombocytopenia. Curr Opin Hematol 2020;27:423–9. 32868673 10.1097/MOH.0000000000000612

[3] VanderVeen N Gupta K Vasquez R. Is thrombocytopenia progressing? Immune thrombocytopenic purpura as warning sign for significant blood disease. Clin Pediatr (Phila) 2020;59:512–5. 31875406 10.1177/0009922819897363

[4] Yung KC Xu CW Zhang ZW. Investigation on glucocorticoid receptors within platelets from adult patients with immune thrombocytopenia. Hematology 2020;25:37–42. 31905108 10.1080/16078454.2019.1710025

[5] Wang H Chen B. Discussion on traditional Chinese medicine treatment of immune thrombocytopenic purpura from blood syndrome. Res Integr Tradit Chin West Med 2020;12:58–60.

[6] Zhao W Qin LK Wang JH. Traditional Chinese medicine's understanding of idiopathic thrombocytopenic purpura. Henan J Tradit Chin Med 2015;35:1451–3.

[7] Liu JJ Du YX Yuan J. Thinking of traditional Chinese medicine syndrome differentiation of idiopathic thrombocytopenic purpura. China Rural Health 2019;11:79.

[8] Zhang B Zhang LN Shang LL. Therapeutic effect of Ziyin Yishen therapy on idiopathic thrombocytopenic purpura and its effects on serum IL-4,IL-6 and immune function in patients with idiopathic thrombocytopenic purpura. Shanxi J Tradit Chin Med 2020;41:194–7.

[9] Jiang ZP. A review on traditional Chinese medicine treatment of immune thrombocytopenic purpura. Chin J Acupunct 2018;7:85–8.

[10] Hou M. Expert consensus on diagnosis and treatment of adult idiopathic thrombocytopenic purpura. Chin J Hematol 2009;30:647–8.

[11] Huang M Li L Zhang XF. Observation on efficacy of Xiandan Shengxue granules in treating idiopathic thrombocytopenic purpura. World Chin Med 2014;9:1307–9.

[12] Kambach S Bruelheide H Gerstner K. Consequences of multiple imputation of missing standard deviations and sample sizes in meta-analysis. Ecol Evol 2020;10:11699–712. 33144994 10.1002/ece3.6806 PMC7593147

[13] Owen RK Bujkiewicz S Tincello DG. Multivariate network meta-analysis incorporating class effects. BMC Med Res Methodol 2020;20:184. 32641105 10.1186/s12874-020-01025-8 PMC7341581

[14] Chan JSK Harky A. The importance of risk of bias assessment in meta-analyses: does controlling heterogeneity suffice? Eur J Cardiothorac Surg 2020;58:1102. 10.1093/ejcts/ezaa174 32776129

[15] Konno K Pullin AS. Assessing the risk of bias in choice of search sources for environmental meta-analyses. Res Synth Methods 2020;11:698–713. 32618107 10.1002/jrsm.1433

[16] Wang Z Lin L Hodges JS. The impact of covariance priors on arm-based Bayesian network meta-analyses with binary outcomes. Stat Med 2020;39:2883–900. 32495349 10.1002/sim.8580 PMC7486995

[17] Wang Q Wang JC Pan B. Advance in the GRADE approach to rate the quality of evidence from a network meta-analysis. Chin J Evid Based Med 2020;20:979–85.

[18] Wang WW Yang ZR Sun F. Development, elaboration and application of grade summary of finding table for network meta-analysis. Chin J Evid Based Med 2020;20:1471–6.

[19] Duan LF. Analysis of the correlation between idiopathic thrombocytopenic purpura and cytomegalovirus and Epstein-Barr virus infection. J Dermatol Ve 2018;40:253–5.

[20] Zhang M Guo B. Use of bioinformatic analyses in identifying characteristic genes and mechanisms active in the progression of idiopathic thrombocytopenic purpura in individuals with different phenotypes. J Int Med Res 2020;48:300060520971437. 33222560 10.1177/0300060520971437 PMC7689594

[21] Yuan YP Yang X Hu BN. Research progress of new drug therapy for adult primary immune thrombocytopenic purpura. J Exp Hematol 2020;28:677–81. 10.19746/j.cnki.issn.1009-2137.2020.02.053 32319415

